# Molecular Dynamics Simulation of Poly(Ether Ether Ketone) (PEEK) Polymer to Analyze Intermolecular Ordering by Low Wavenumber Raman Spectroscopy and X-ray Diffraction

**DOI:** 10.3390/polym14245406

**Published:** 2022-12-10

**Authors:** Xiaoran Yang, Seiya Yokokura, Taro Nagahama, Makoto Yamaguchi, Toshihiro Shimada

**Affiliations:** 1 Graduate School of Chemical Science, Hokkaido University, Kita 13 Nishi 8, Kita-ku, Sapporo 060-8628, Japan; 2 Division of Applied Chemistry, Faculty of Engineering, Hokkaido University, Kita 13 Nishi 8, Kita-ku, Sapporo 060-8628, Japan; 3 Department of Systems Design Engineering, Akita University, 1-1 Tegatagakuen-machi, Akita 010-8502, Japan

**Keywords:** poly(ether ether ketone) (PEEK), molecular dynamics simulation, low wavenumber Raman spectroscopy

## Abstract

Poly(ether ether ketone) (PEEK) is an important engineering plastic and evaluation of its local crystallinity in composites is critical for producing strong and reliable mechanical parts. Low wavenumber Raman spectroscopy and X-ray diffraction are promising techniques for the analysis of crystal ordering but a detailed understanding of the spectra has not been established. Here, we use molecular dynamics combined with a newly developed approximation to simulate local vibrational features to understand the effect of intermolecular ordering in the Raman spectra. We found that intermolecular ordering does affect the low wavenumber Raman spectra and the X-ray diffraction as observed in the experiment. Raman spectroscopy of intermolecular vibration modes is a promising technique to evaluate the local crystallinity of PEEK and other engineering plastics, and the present technique offers an estimation without requiring heavy computational resources.

## 1. Introduction

Poly(ether ether ketone) (PEEK) is a high-performance thermoplastic and crystalline polymer with excellent mechanical properties, inertness in a harsh chemical environment, and thermal durability [1,2,3]. Reinforced composite materials of PEEK with inorganic or carbon fiber fillers have attracted attention as structural materials for aircraft and automobiles because of their light weight and excellent properties. It is established that the degree of crystallinity greatly affects the mechanical properties of crystalline polymers including PEEK. Since it has been reported that the crystallinity of fiber-reinforced polymers may be spatially non-uniform [4,5], it is important to evaluate local crystallinity less than the diameter of the filler fibers. Since the size of the fiber fillers is on the scale of several μm, evaluation of crystallinity in the sub-μm region is important.

There are various techniques for the evaluation of the crystallinity of polymers such as wide-angle X-ray diffraction (WAXRD), small-angle X-ray diffraction (SAXS), differential scanning calorimetry (DSC), Fourier transform infrared (FTIR) spectroscopy, and Raman microscopy [6,7]. Among them, Raman microscopy, which takes Raman spectra under the focus of an objective lens, is probably the only readily available technique [8]. X-ray diffraction microscopy using a synchrotron light source [9] or cryogenic transmission electron microscopy [10] is now possible, but the location and machine time for the measurement are very limited and preparation of the samples in very thin slabs is troublesome. Raman microscopy can be measured in the reflection geometry with the high resolution of optical microscopy that can easily reach sub-μm and possibly measure the local vibrational spectrum.

There are several reports on the application of Raman spectra for the evaluation of the crystallinity of PEEK [6,7,11,12,13,14,15]. Usually, the frequency shift of fundamental vibration modes involving a single or several covalent chemical bonds (*intra*molecular modes) is used to characterize the crystallinity. However, the shift of the intramolecular modes comes from the difference in the local environment surrounding the chemical bonds, and it does not reflect the crystallinity straightforwardly. There are also “*inter*molecular modes” in molecular materials, including polymers, which reside at low frequency [16,17,18]. Intermolecular modes are expected to be more sensitive to the crystallinity, because the intermolecular distances and angles and chain alignment in polymers will be affected by the crystallinity. A team including some of the present authors has reported the use of a low-frequency Raman signal to evaluate the crystallinity of PEEK [7]. Although they found that the intensity of a Raman signal changed during crystallization as explained in the next section, the mechanism has not been elucidated. This paper aims to understand the mechanism from molecular dynamics simulation (MD) with a new analysis scheme.

It is now possible to calculate the Raman spectra from first principles using the fragment method [17] and plane wave methods. Although the accuracy of the calculation for polymers has been established in comparison with experiments [18,19], the calculation of the Raman spectra requires a quantitative evaluation of the electronic structure of the material and its change caused by vibration. It is computationally very heavy to be applied to a large inhomogeneous system including polymer composites, which are important in practical applications.

In this paper, we present a computationally alleviated approach using classical MD. MD can track the motion of each atom under various conditions, such as constant temperature, pressure, volume, energy, etc. The information on the chemical bonding and intermolecular interaction is contained in the force field (FF) in the classical MD. There are various types of FF and the parameters have been determined by various experimental physical properties such as melting points and heat of phase transitions, and some of them use the result of quantum chemical calculations. They are still improved by using first-principle calculations combined with a data science approach. If the MD is performed for a long enough time and the atomic motion is “Fourier-transformed”, it should contain the information on molecular/lattice vibrations. It will be a reasonable alternative for molecular vibration analysis if the atomic motion can be related to spectroscopy such as Raman spectroscopy. We here propose an approximation of the local polarizability by approximated valence electron densities that will be explained in the Materials and Method section. Fast Fourier transform (FFT) of the time variation of the local polarizability is used to derive data corresponding to Raman spectra. Intramolecular vibration is evaluated by DFT quantum chemical calculation.

The MD can be used to simulate X-ray diffraction as well, which is more straightforward than the vibrational analysis and implemented in many software packages. We use it to compare the experimental wide-angle X-ray diffraction (WAXRD).

## 2. Materials and Methods

### 2.1. Extraction of Approximate Raman Signal from MD Simulation

The probability of Raman scattering is described by the Raman polarizability tensor, which is simplified by Placzek approximation [20,21] to Equation (1):

(1) $${\left(\alpha_{\alpha \beta }\right)}_{v_{m}v_{n}}=\frac{2}{\hbar }\underset{j\neq n}{\sum }\frac{\omega_{jn}}{\omega_{jn}^{2}-\omega^{2}}\langle v_{m}|Ren|\mu_{\alpha }|j\rangle \langle j|\mu_{\beta }|n|v_{n}\rangle$$

in the case of Stokes Raman scattering. Here, the lefthand side of the equation is the Raman polarizability tensor corresponding to the vibrational transition from $v_{n}$ to $v_{m}$, and ***n*** and ***j*** denote the ground state of electrons and excited states, respectively. ***ω_jn_*** is the energy difference between states ***n*** and ***j*** in angular frequency and ***ω*** the angular frequency of the incoming electromagnetic wave. $\mu_{\alpha }$ and $\mu_{\beta }$ are dipole moment operators in the direction of ***α*** and ***β***.

Calculation of this quantity requires the calculation of precise electronic states of the molecule and its dependence on the position of the nuclei vibrating from the ground state. It becomes computationally very demanding in the case of polymer aggregates because of the many degrees of structural freedom as mentioned in the Introduction. Also, accurate treatment of van der Waals interactions becomes computationally very demanding but necessary for the estimation of intermolecular vibration. Our approach proposed here is to use an approximate local valence electron density. The motion of valence electrons by the external electric field is the cause of the dynamic dipole moment associated with the transition $\langle n|\mu_{\alpha }|j\rangle$ and $\langle j|\mu_{\alpha }|n\rangle$. The fluctuation of the local valence electron density at the frequency of a vibration mode $v_{m}$ can be assumed proportional to them. Therefore, we can approximate the Raman intensity at vibrational frequency ***ν*** as proportional to the squared oscillation of valence electron density ρ caused by vibration:

(2) $${\left(\alpha_{\alpha \beta }\right)}_{v_{m}v_{n}}\propto \rho^{2}\left(\nu \right)$$

Since it is established that Raman scattering can occur in a single molecule [22], we can justify a crude approximation to neglect the spatial phase of the electric field of the incoming photons and consider the scattering locally at position *x* in the material:

(3) $${\left(\alpha_{\alpha \beta }\right)}_{v_{m}v_{n}}\;\propto \int \rho^{2}\left(x,\nu \right)dx$$

There will be various ways to calculate the local valence electron density. In this paper, we use a very simple approximation as follows:

(4) $$\rho \left(x\right)=\underset{i}{\sum }N_{i}exp(-\frac{\left|x-r_{i}\right|}{R_{i}})$$

where ρ (***x***) is the valence electron density at position ***x*** and *N*_i_ and *R*_i_ are the valence electron number and characteristic atom radius of atom *i*, respectively. ***r***_i_ is the position of atom *i*. Here we used values for *N*_i_ the nominal valence electron count of each atom (C 4, O 6, H 1), and for *R*_i_ covalent radii were used (C 0.73 Å, O 0.66 Å, H 0.32 Å) [23]. In the following, we show the Fourier transform of ρ, not ρ^2^, to show the small features in the figures. The conclusion does not change if we use ρ^2^.

This type of simplification of molecules has recently been used in machine learning for protein engineering and has been proven to work to extract the microchemical environment of biological polymers [24,25].

We must admit that the crude approximation in Equation (3) neglects the shape and symmetry of the wave functions of the molecule or its fragment, and the “spectrum” obtained by the Fourier transform of Equation (4) contains both infrared absorption and Raman scattering. The merit of doing this is the short computational time for large systems. We expect the usage of the present method is calculating large systems to find the local environment that causes a certain Raman signal (or infrared absorption). Then we can proceed with the first-principle calculation of extracted local structures to accurately evaluate it.

### 2.2. Computation Details

Molecular vibrations of single-strand PEEK oligomers were calculated with DFT calculation using Gaussian16 [26] at B3LYP/6-31G level of theory. The MD simulation was performed using LAMMPS [27] with generalized AMBER FF (GAFF) [28]. The atomic charges for the calculation of Coulomb interactions were calculated by Gaussian16 of a PEEK oligomer at B3LYP/6-31G level. The initial structure of the polymer based on the reported crystal structure [29] was prepared using the moltemplate utility [30]. There are several crystal structure parameters for PEEK [31,32,33,34,35,36,37,38] but they agree with each other only with a small deviation of the lattice constant and atom positions [6]. The results were visualized with OVITO [39] and VESTA [40]. The atomic trajectory data, including Fourier transform and smoothing spline fitting, were analyzed using programs written with Python 3.7. The vibration mode at frequency ***ν*** (Hz) is expressed in the unit of wavenumber $\tilde{\nu }$ (cm^−1^) throughout this paper using

(5) $$\tilde{\nu }=\nu /c$$

where *c* is the light speed.

### 2.3. Experiments

To compare the calculation with the experiments, WAXRD patterns and Raman spectra were measured on the samples annealed at various temperatures up to 300 °C for 2 h. The sample material was a quenched PEEK thin film with a thickness of 50 mm (Victrex APTIV film, grade 1000). Details of the experiment are described in [7]. WAXRD was measured by using Rigaku Ultima IV (rotating anode, 40 kV 30 mA) operating with CuKa X-ray (wavelength 1.5406 Å). Raman spectroscopy was measured using a Horiba Jobin Yvon LablamHR spectrometer with a Nd:YAG laser (wavelength 1064 nm, 60 mW, spot size 1 μm) with a 50× magnification microscope. Signals from a 10 s measurement were accumulated four times to confirm no damage to the sample during the measurement.

## 3. Results and Discussion

### 3.1. Experimental Results

Before presenting computational analysis, the experimental findings are reviewed briefly. The experimental data have been reported previously [7] but here we newly processed the data in a form suitable for comparison with the computational results.

**Figure 1:**
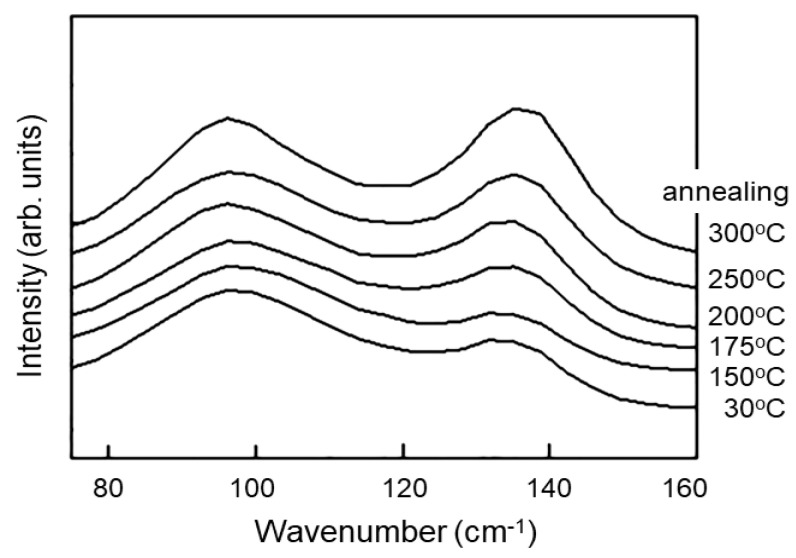
Low wavenumber Raman spectra of PEEK films annealed at various temperatures (measurement at room temperature).

**Figure 2:**
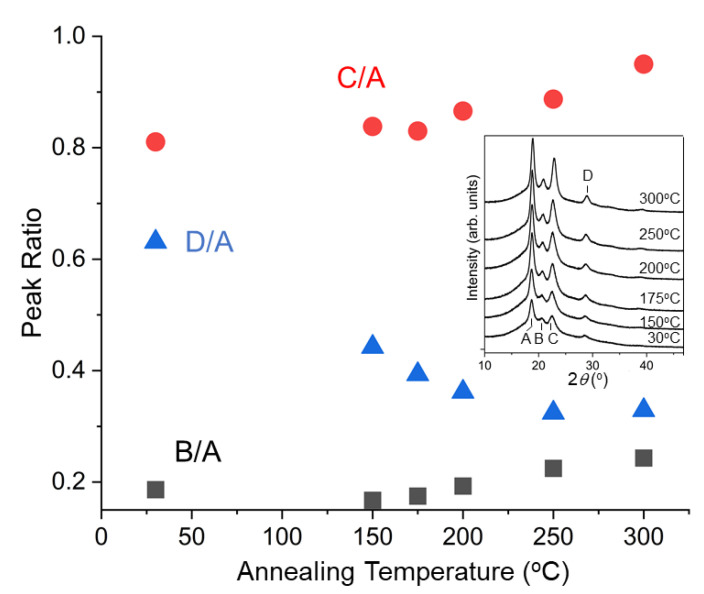
Area ratios of WAXRD peaks B (2*θ* = 20.7 °), C (22.8 °), D (28.8 °) to peak A (18.9 °) calculated after removing the broad background coming from the amorphous portion. The inset shows the raw WAXRD spectra.

Figure 1 shows Raman spectra of PEEK after annealing at various temperatures for two hours. It can be noticed that there are two peaks in this low wavenumber region at 97 cm^−1^ and 135 cm^−1^, and the intensity of the 135 cm^−1^ peak increases whereas that at 97 cm^−1^ does not change significantly. Figure 2 shows WAXRD patterns (inset) and the peak ratios of the same samples. There are four distinct peaks (Peak A 2*θ* = 18.9°, B 20.7°, C 22.8°, D 28.8°), and the peak ratio changes by annealing at different temperatures. These features in Raman and WAXRD can be used for the evaluation of the crystallinity of PEEK, but the mechanism of the change has not been understood so far. In the following sections, we use computational methods to understand these features.

### 3.2. Calculated Raman Spectroscopy of a Single Molecule PEEK Chain

**Figure 3:**
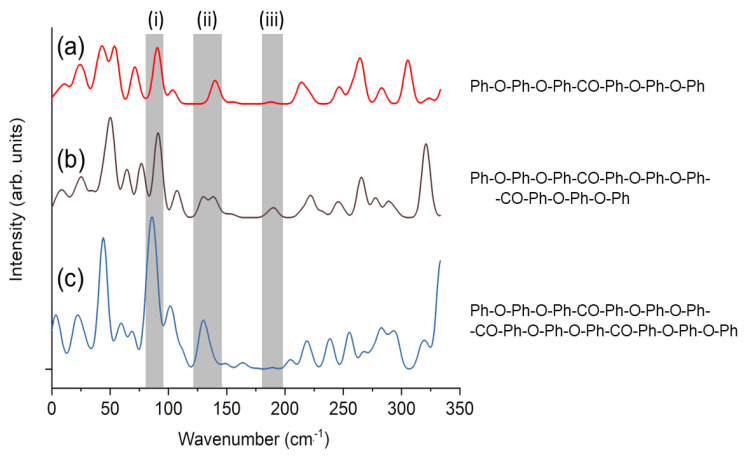
DFT-calculated Raman spectra of phenyl-terminated PEEK oligomers shown on the right. The peaks around 85–98 cm^−1^, 130–145 cm^−1^ and 190 cm^−1^ labeled as (i), (ii), (iii), respectively. (**a**–**c**) represent different lengths of a PEEK molecule chain, the structures of molecules are written in the right panel.

**Figure 4:**
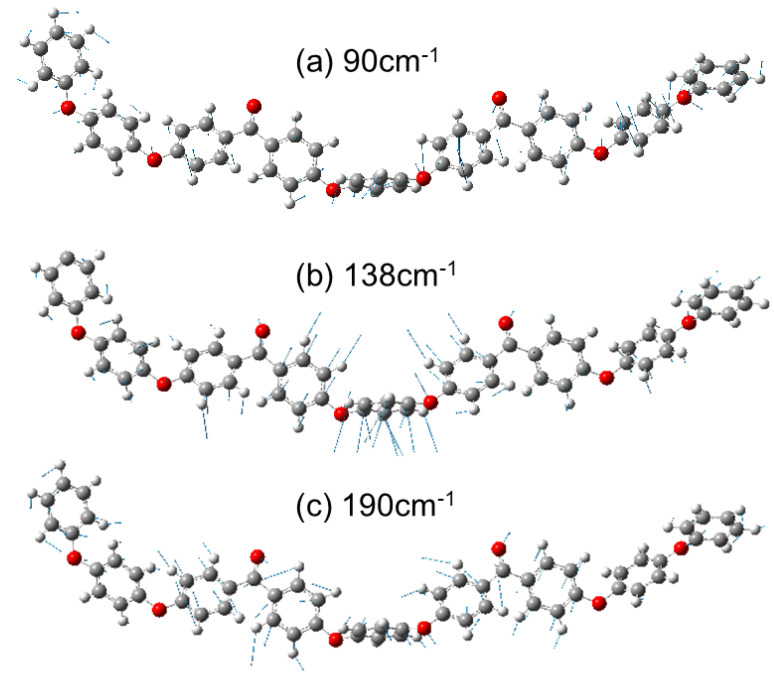
Atomic motion in the vibrational modes at (**a**) 90 cm^−1^, (**b**) 138 cm^−1^ and (**c**) 190 cm^−1^, calculated for a single oligomer molecule by DFT.

In order to understand the vibration of a PEEK molecule, DFT (b3lyp/6-31G) optimization and calculation of Raman intensity of each vibrational mode of phenyl-group-terminated PEEK oligomers with different lengths were performed using the Gaussian 16 package. The results are shown in Figure 3 and Figure 4. Figure 3 shows the Raman spectrum derived from the Raman intensity of each mode with 5 cm^−1^ broadening. The structures of the molecules are written in the right panel, in which “Ph”, “–O–” and “–CO–” denote phenyl group (C_6_H_5_– or –C_6_H_4_–), ether, and ketone, respectively. The peaks around 85–98 cm^−1^ (labeled as “(i)”) and 130–145 cm^−1^ (“(ii)”) are those corresponding to the experimental observation (97 cm^−1^ and 135 cm^−1^). The scaling factor for vibrational analysis using DFT (b3lyp functional) is 0.961 (only ~4% difference) [41] and can be neglected because the shift of the modes due to the chain elongation is larger than 4%.

Gaussian peaks with 5 cm^−1^ broadening were summed up after multiplying the calculated Raman intensities.

By scrutinizing the atomic motion of each mode (Figure 4), these modes were roughly identified as follows: The 85~98 cm^−1^ (mode (i)) is scissoring of ether (Ph–O–Ph) moiety and the 130~145 cm^−1^ (mode (ii)) is twisting of ketone and connected benzene rings (Ph–CO–Ph). In Figure 4, the molecule corresponding to Figure 3b is shown. The shorter molecule in Figure 3a has the vibration mode corresponding to Figure 3a,b at 90 cm^−1^ and 138 cm^−1^, respectively. For the longer molecule in Figure 3c, these modes are split into several modes, i.e., {83, 86, 89 cm^−1^} and {128,131,135 cm^−1^}, due to the lowered symmetry of the chain after optimization as a single molecule. The Raman active vibration at 190 cm^−1^ appearing as a weak peak (iii) in Figure 3b is also displayed in Figure 4c, because it is observed in the simulation as explained later. This mode is related to the rotation around ketone and becomes lowered (164–189 cm^−1^) when the molecule becomes longer (Figure 3c).

### 3.3. MD Simulations

**Figure 5:**
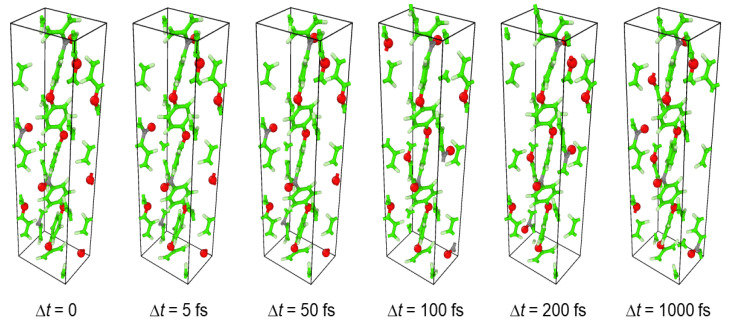
Snapshots of MD simulation of a unit cell of PEEK under NPT ensemble at 300 K and 1 atm.

The MD simulation was performed using LAMMPS with GAFF and a 0.1 fs time step. The atomic trajectory was recorded at 5 fs intervals. It was performed under NPT constraint, in which the number of particles (N) was constant and the pressure (P) and temperature (T) were controlled to be certain values with feedback. The following numerical analysis including temporal Fourier transform can be performed in several minutes. It is much shorter than the vibrational analysis of a polymer system using other techniques. Figure 5 shows snapshots from the MD trajectory at 300 K and 1 atm. The video can be found in supplement, Video S1 (time interval corresponding to 90 cm^−1^) and Video S2 (135 cm^−1^).

### 3.4. Local Vibrational Spectra Simulated from MD

**Figure 6:**
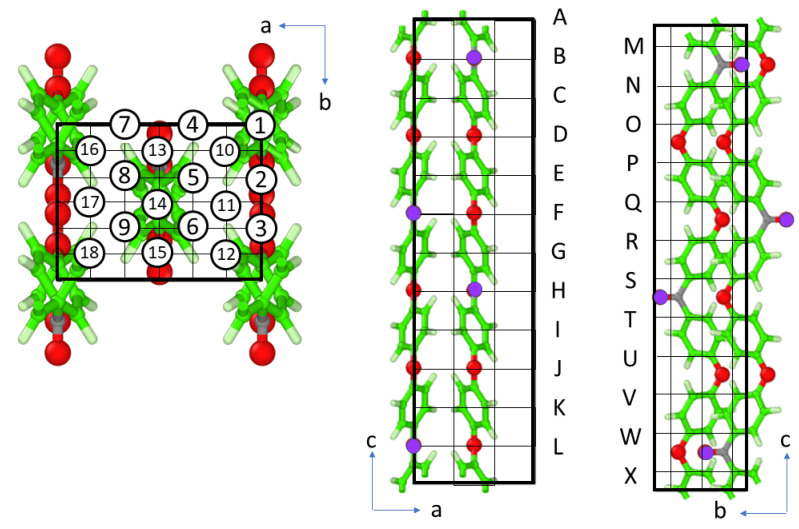
Unit cell of PEEK polymer and notation of the calculated points. *a*, *b*, and *c* are unit cell directions. ①~⑨ (⑩~⑱) in *ab* plane were coupled with A~L (M~X) in the *c* direction. A point is denoted with a circled number and an uppercase letter in the figure. Aromatic carbons are green, hydrogens are white, ether oxygens are red, and ketone carbons and oxygens are shown in purple.

The trajectory of atoms from the MD simulation was used to calculate the local valence electron densities (ρ) at a 5 fs interval. The 2 × 3 × 3 × 12 (=216) points were selected in the unit cell and ρ of each point was calculated as a function of time using Equation (4). The points are identified using two characters as shown in Figure 6: circled numbers (1)~(18) in the *ab* plane and letters A~L and M~X in the *c* direction.

**Figure 7:**
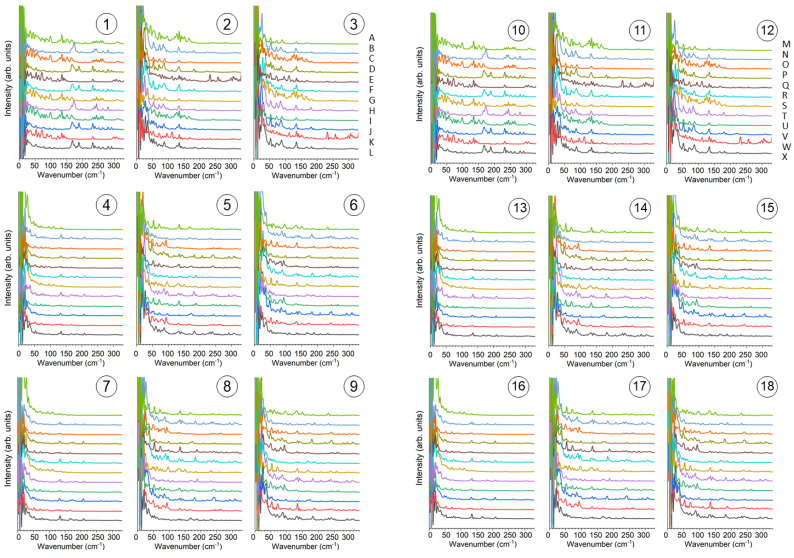
Simulated local vibrational feature of “valence electron density” of PEEK obtained from Fourier transform of approximate local valence electron density calculated from the atomic trajectory in MD. The curves in each figure from top side to bottom side correspond to the calculated points in each column from top side to bottom side in *c* direction. The positions of calculated points can be found through a circled number and an uppercase letter as we introduced in Figure 6.

Circled (1)~(9) in the *ab* plane were coupled with A~L in the *c* direction to make 9 × 12 points, and circled (10)~(18) were coupled with M~X to make another 9 × 12 points. The local vibrational features were calculated as follows. Approximated “valence electron density” was obtained by Equation (4) using an atomic coordinate snapshot of 300 K 1 atm NPT simulation recorded at 5 fs intervals. The sum of Equation (4) at each position (3 × 3 × 12 (=108) positions (①A~⑨L)) was taken over the atoms in the same unit cell and nearest-neighboring unit cells (total 3 × 3 × 3 = 27) using a cut-off distance of 10 Å. A time duration of 45 ps after reaching thermal equilibrium was used for the calculation. The approximated “valence electron density” had 3 × 3 × 12 array data with 9000 points. They were Fourier-transformed and the absolute values were smoothed by smoothing-cubic spline. The results are shown in Figure 7 after converting the frequency axis to the wavenumber unit using Equation (5). The scaling factor for the vibration frequency derived from the MD using GAFF is reported to be 1.0 [41] and there is no need for scaling the frequency.

**Figure 8:**
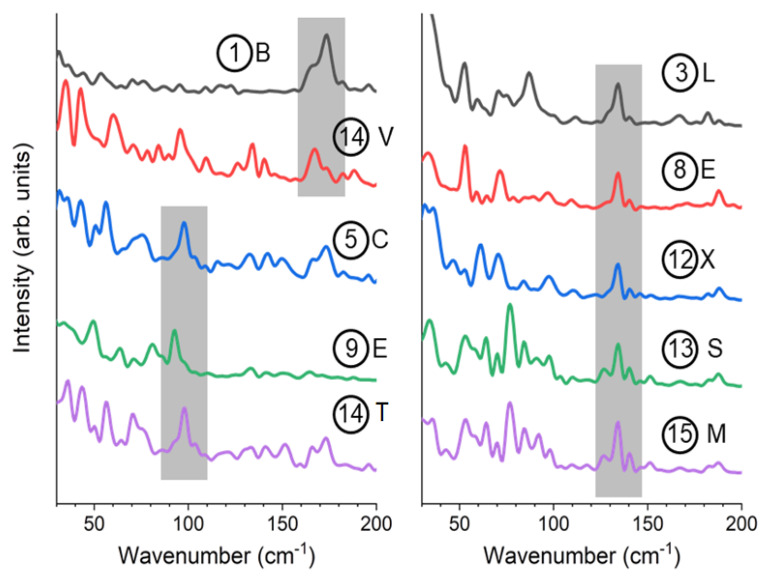
Simulated spectra showing characteristic peaks selected from Figure 7. The number in the circle and the uppercase letters correspond to those shown in Figure 6. Colors of the curves are to distinguish the curves and do not have specific meaning. The 175 cm^−1^ and 90 cm^−1^ peaks are hatched in gray in the left panel, and the 135 cm^−1^ peaks are hatched in the right panel. Circled numbers and letters show the point of calculation.

We notice that several prominent peaks are observed in the low-frequency region in Figure 7. Typical ones are picked up and magnified in Figure 8. It is noted that 175 cm^−1^ is very local in some positions in column ① (Figure 8 top left), which coincides with the positions of ketone moiety. Column ⑭located at the center of the molecular chain also shows 175 cm^−1^ peaks. Vibrations of 90 cm^−1^ (Figure 8 left) and 135 cm^−1^ (Figure 8 right) are observed more often at the intermolecular spaces (⑤⑨③⑧⑫) than in center of the molecular chain (①⑭).

The results presented here show that Fourier transform of the “valence electron density” (Equation (4)) reproduces the precise vibrational analysis by DFT and the experimental observation. Considering that the 135 cm^−1^ Raman signal is much weaker than that of 90 cm^−1^ in DFT of single molecules, and the MD combined with Equation (4) gives prominent peaks at 135 cm^−1^ at the intermolecular positions, we can presume that the 135 cm^−1^ mode is strongly affected by intermolecular interaction. This identification is consistent with the different behavior of 90 cm^−1^ and 135 cm^−1^ peaks during the improvement of crystallinity by the annealing experiment.

We must admit that there are some insufficient points in the present analysis of Raman spectra—the 175 cm^−1^ peak found in the analysis was not experimentally observed. It may be explained by the selection rule considering the phase factor of the electric field of light. However, the result can be obtained with much fewer computational resources compared with first-principle techniques that are difficult to apply to complicated crystalline polymers and composites.

### 3.5. WAXRD Pattern from MD Simulation

**Figure 9:**
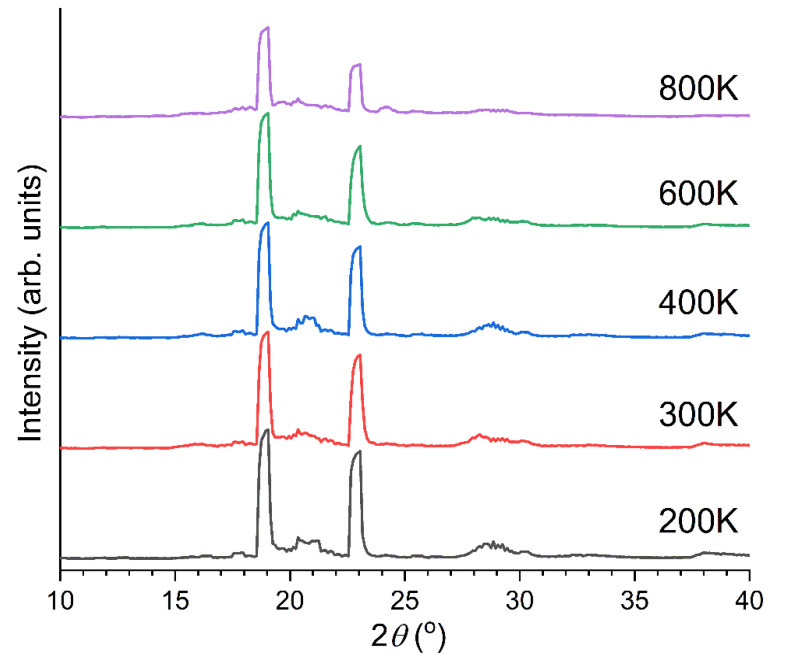
WAXRD patterns simulated using LAMMPS for a PEEK oligomer supercell corresponding to 9 × 9 × 5 unit cells.

X-ray diffraction calculation was implemented in LAMMPS. We used this feature to explain the experimental results of WAXRD. First, we used large supercells of infinite polymers, but the structure did not change even when the simulation temperature was raised to 1000 K. We considered the periodic boundary condition (PBC) with connected covalent bonding to be too strong a restriction. Next, we tried to remove the PBC and run an NPT simulation. The structure soon became amorphous when the temperature was raised beyond 400 K, and crystallization was not observed even though the system was cooled on the scale of tens of ns. This is because the in- silico crystallization requires time beyond ordinary computation in the case of polymer simulation [42]. We therefore prepared crystalline phenyl-terminated oligomers corresponding to 9 × 9 × 5 unit cells and set PBC to the whole supercell to run the NPT calculation. Then the WAXRD patterns from the simulation changed according to the temperature. The results are shown in Figure 9. Still the PBC has an influence and the crystalline order is kept beyond the melting point of PEEK. However, the peak intensities diminish when the temperature is raised. We believe that this result can be used to study the effect of the degree of disorder on the WAXRD.

**Figure 10:**
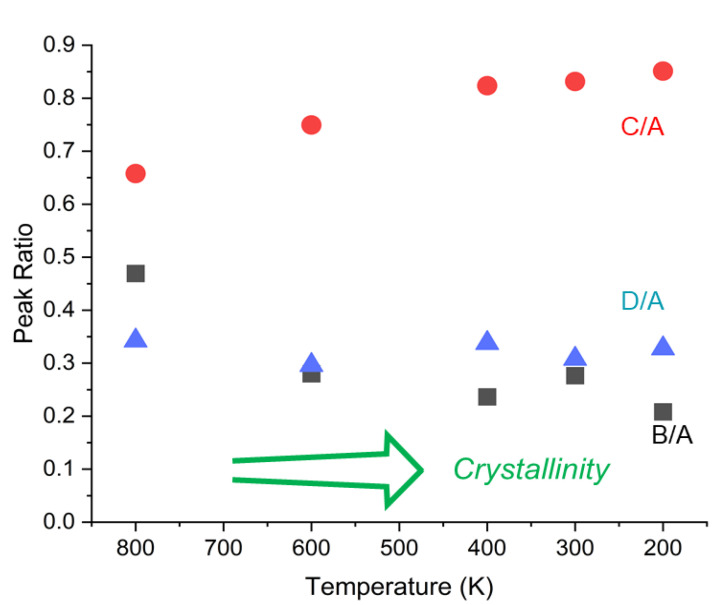
The ratio of peak areas as a function of temperature in the simulation. Area ratios of peaks B, C, D to peak A labeled same as experimental results (Figure 2).

In Figure 2, the initial state of the sample was mainly amorphous obtained by quenching and the crystallinity became improved as the annealing temperature increased with a certain time step. Since the simulation temperature corresponds to thermal disorder in the supercell, it is reasonable to compare the peak area as a function of temperature in the reverse direction as shown in Figure 10. It is noted that peak ratio C/A increases when the crystallinity is increased. This agrees well with the experimental result shown in Figure 2. Peaks A and C correspond to 110 and 200 diffractions, respectively. The result can be explained by considering the order of the nearest neighbor (110) to be first established and that of the next nearest neighbor (200) to follow to improve when the crystallinity is enhanced during annealing. Peaks B and D correspond to 111 and 211 diffractions. We believe the effect of PBC works strongly in the *c*-direction (the third coordinate) because the supercell did not exist in this direction and thus discrepancy with the experiment is observed for these peaks.

### 3.6. Future Improvement of the Present Method

We have tried to extract information on intermolecular vibration using a concept used in machine learning of microchemical environments. It is applied to the classical MD trajectory of PEEK crystal and it shows agreement with the experiment. However, we admit that the estimation of the Raman intensity of each mode requires a more accurate estimation. It is desired to combine the MD with quantum chemical techniques, and some research in this direction has been reported on quantum MD [43,44]. The reported methods still require heavy computational resources and cannot be applied to large systems. For example, the use of machine learning techniques to identify the position and direction of Raman-sensitive chemical moieties will be the direct extension of the present work.

## 4. Conclusions

We have proposed and demonstrated a new analysis procedure for MD time trajectory to derive approximated Raman spectra in the low-frequency region. It involves the local fluctuation of “valence electron density” as a function of time, which is converted to a vibrational spectrum by the Fourier transform. From the calculation of PEEK polymer, 90 cm^−1^, 135 cm^−1^, and 175 cm^−1^ peaks were observed, among which 90 cm^−1^ and 135 cm^−1^ peaks were experimentally observed. The 135 cm^−1^ signal was strongly observed at the intermolecular point, and it is suggested that it is strongly influenced by intermolecular interaction. This explanation agreed well with the experimental behavior of the peak intensity during crystallization. The WAXRD experiment was compared with the MD with different crystallinity at different temperatures, and nearest-neighbor ordering is more quickly established than that involving the next-nearest neighbors. Although there is room for improvement, the present technique provides a convenient way to estimate the vibrational spectra of polymers and their composites with much fewer computational resources than ordinary techniques.

## Data Availability

MD trajectory data can be provided upon request.

## Supplementary Materials

The following supporting information can be downloaded at: https://www.mdpi.com/article/10.3390/polym14245406/s1, Video S1: MD snapshots corresponding to vibration at 90 cm^−1^. Available online: https://youtu.be/8yChaA3DBxE (accessed on 6 December 2022); Video S2: MD snapshots corresponding to vibration at 135 cm^−1^. Available online: https://youtu.be/cq8OaKman78 (accessed on 6 December 2022).
